# Use of Empirical Mode Decomposition in ERP Analysis to Classify Familial Risk and Diagnostic Outcomes for Autism Spectrum Disorder

**DOI:** 10.3390/brainsci11040409

**Published:** 2021-03-24

**Authors:** Lina Abou-Abbas, Stefon van Noordt, James A. Desjardins, Mike Cichonski, Mayada Elsabbagh

**Affiliations:** 1 Montreal Neurological Institute, McGill University, Montreal, QC H3A 2B4, Canada; stefonv0@gmail.com (S.v.N.); mayada.elsabbagh@mcgill.ca (M.E.); 2 Cognitive and Affective Neuroscience Lab, Brock University, St. Catharines, ON L2S 3A1, Canada; james.desjardins@computeontario.ca (J.A.D.); mike.cichonski@gmail.com (M.C.)

**Keywords:** autism spectrum disorder, event-related potential, empirical mode decomposition, intrinsic mode functions, support vector machine, *k*-nearest neighbor

## Abstract

Event-related potentials (ERPs) activated by faces and gaze processing are found in individuals with autism spectrum disorder (ASD) in the early stages of their development and may serve as a putative biomarker to supplement behavioral diagnosis. We present a novel approach to the classification of visual ERPs collected from 6-month-old infants using intrinsic mode functions (IMFs) derived from empirical mode decomposition (EMD). Selected features were used as inputs to two machine learning methods (support vector machines and *k*-nearest neighbors (*k*-NN)) using nested cross validation. Different runs were executed for the modelling and classification of the participants in the control and high-risk (HR) groups and the classification of diagnosis outcome within the high-risk group: HR-ASD and HR-noASD. The highest accuracy in the classification of familial risk was 88.44%, achieved using a support vector machine (SVM). A maximum accuracy of 74.00% for classifying infants at risk who go on to develop ASD vs. those who do not was achieved through *k*-NN. IMF-based extracted features were highly effective in classifying infants by risk status, but less effective by diagnostic outcome. Advanced signal analysis of ERPs integrated with machine learning may be considered a first step toward the development of an early biomarker for ASD.

## 1. Introduction

Autism spectrum disorder (ASD) is a complex and heterogeneous condition that affects communication, social interaction, and behavior. According to the World Health Organization, approximately 1 in 160 children has a diagnosis of ASD. There appear to be genetic and familial risk factors that increase the likelihood of ASD, given that nearly 20% of infants who have an older sibling with an ASD diagnosis eventually receive a diagnosis of ASD themselves [1]. Based on this increased risk, there has been growing interest in research studies focused on infants with ASD-affected siblings (high risk; HR). These infant-sibling studies are critical for examining prospective developmental markers of ASD and improving early detection prior to the emergence of behavioral symptoms. Several of these studies implicate atypical early brain development [2,3,4] that precedes the appearance of core behavioral symptoms that reflect the broader autism spectrum [5,6].

Electroencephalogram (EEG) is the most widely used tool for measuring brain function due to its being noninvasive, efficient in acquisition, and relatively low cost. Studies on the early functional markers of ASD have shown that atypical brain responses during the processing of faces and dynamic changes in eye gaze direction are found in children with ASD and infants who are at risk for ASD [2,6,7,8]. These early studies suggest that atypical brain responses to faces and eye gaze may serve as putative markers that reflect increased risk and could supplement behavioral diagnosis of ASD.

In this regard, the neural markers of gaze and face processing in ASD have been extensively investigated using event-related potentials (ERPs) [2,6], which reflect systematic changes in the scalp-recorded EEG that are time-locked and phase-locked to the onset of a stimulus (e.g., face) or behavioral response (e.g., button press) [9]. Previous studies on HR infants have documented clear differences in several ERP components that are elicited during face and gaze processing. Specifically, compared with typically developing 10-month old infants, HR infants show a slower response, as reflected by peak latency, in both the N290 and P400 components when processing faces versus objects [10]. Similarly, other studies suggest that the P400 component to gaze direction (direct/averted) and face familiarity (familiar/stranger face) also distinguishes HR from typically developing infants [6,11]. Beyond testing group differences on a relatively small number of factors (e.g., P400 latency), the field of machine learning has shown promise in various biomedical applications, such as computer-aided diagnosis through mining multivariate data. These approaches can aid the interpretation of physiological data and be used by clinicians to consider disease diagnosis based on multiple sources of information (e.g., EEG, electrocardiography, electronic medical records, and magnetic resonance imaging). There has been growing interest in investigating ASD diagnostic utility through neuroimaging data using machine learning [12,13,14,15,16].

Only a few studies have been conducted using machine learning analyses combined with behavioral measures [4,14,17,18]. In one study [4], machine learning was applied using the Mullen Scales of Early Learning and Vineland Adaptive Behavior Scales at 8 months and showed an accuracy rate of 69.2% in predicting ASD outcomes. In a separate study [17], a support vector machine (SVM) classifier was used to differentiate ASD individuals based on the Autism Diagnostic Interview (ADI) and Social Responsiveness Scale (SRS), showing a sensitivity of 89.2%. In terms of ERPs, only one study has applied machine learning techniques to distinguish HR from typically developing infants at 6 months of age [19]. Despite the use of multiple machine learning methods and multiple features across experimental conditions, the highest accuracy rate obtained was only 64%.

ERP data analysis has been largely restricted to fixed-latency and amplitude-based differences between experimental conditions or groups, which may obscure the richness of EEG features that are relevant for face processing and may be linked to risk and diagnostic outcomes. Although it is also possible to decompose ERPs into their time-varying spectral features in order to extract more nuanced measures of brain function, no studies to date have investigated the effect of ERP transformation on time and/or frequency domains in combination with machine learning.

The current study examined whether using features from transformed ERPs as input can improve the accuracy of classification of family risk and diagnostic outcome. We chose to apply the empirical mode decomposition (EMD) technique, which is an adaptive data processing method designed for linear and stationary signals. The EMD technique preserves the frequency variation in time and has been widely used in the domain of biomedical signals [20,21,22,23]. In the current study, we propose a novel approach to classify ERPs’ generated static faces and dynamic eye gaze using intrinsic mode functions (IMFs). Specifically, the EMD technique was used to decompose ERPs into a set of IMFs, from which six quantitative features were calculated (IMF energy, Shannon entropy, and four statistical parameters). These quantitative features were used to assess the classification accuracy across different machine learning algorithms.

## 2. Method

### 2.1. Participants

The current sample comprised 104 infants from the British Autism Study of Infant Siblings (BASIS) study. EEG data were collected from 54 infants at HR for autism by virtue of having an older diagnosed sibling or half-sibling, and 50 control infants. EEG collection occurred when infants were 6 to 10 months of age (mean = 7.8 months, SD = 3.72). A diagnosis of ASD was confirmed in a subgroup of participants when they reached 24 months and 36 months of age based on clinical research scores that included the Autism Diagnostic Observation Schedule (ADOS) and the Autism Diagnostic Interview (ADI). Ten participants were excluded due to insufficient ERP data (specified as fewer than 10 trials per condition; *n* = 6) and/or lack of information regarding diagnostic outcome (*n* = 4). The final sample included 17 HR infants who subsequently received a diagnosis (HR-ASD), 33 high-risk infants who did not receive a diagnosis (HR-noASD), and 44 control infants. Table 1 lists a descriptive summary of the sample size and biological sexes for the three groups.

**Figure 1:**
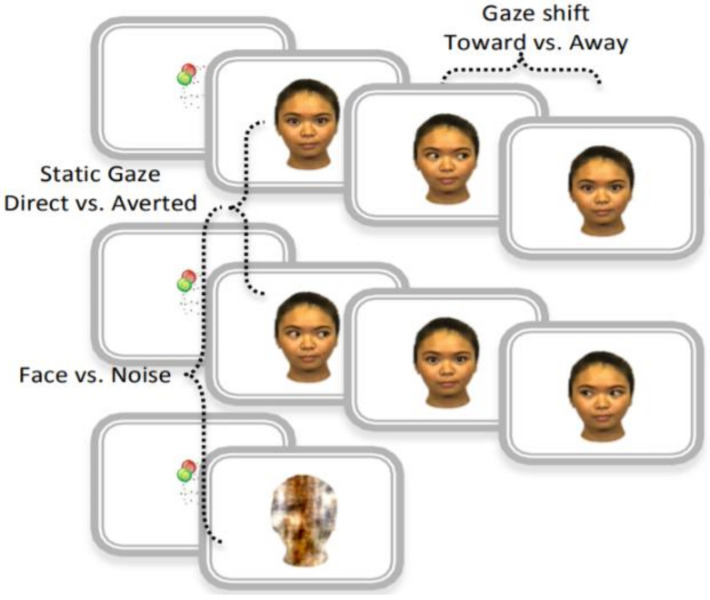
Event-related potential (ERP) task corresponding to visual stimulus used in the original study: static gaze—direct and averted. Face and noise—gaze shift toward and away- Reprinted from ref. [6] (Supplemental Data Section Figure S2).

A 128-channel HydroCel Geodesic Net was used to collect EEG signals digitized at 500 Hz. Event-related potentials (ERPs) were recorded in response to different face stimuli, illustrated in Figure 1: static gaze condition (direct versus averted), dynamic gaze condition (toward versus away), and face condition (including any static or dynamic gaze condition versus noise). The face stimuli consisted of four different female faces, each with three different gaze directions (direct or averted to the left or right). All faces contained a neutral expression. The infants were seated approximately 60 cm from the presentation screen, which was 40 × 29 cm in size. The faces subtended 21.3 × 13.9 degrees, with eyes subtended approximately 1.6 × 2.7 degrees. For more details on data collection and clinical assessments, see [6] (Supplemental Data Section).

### 2.2. Analysis Framework

**Figure 2:**
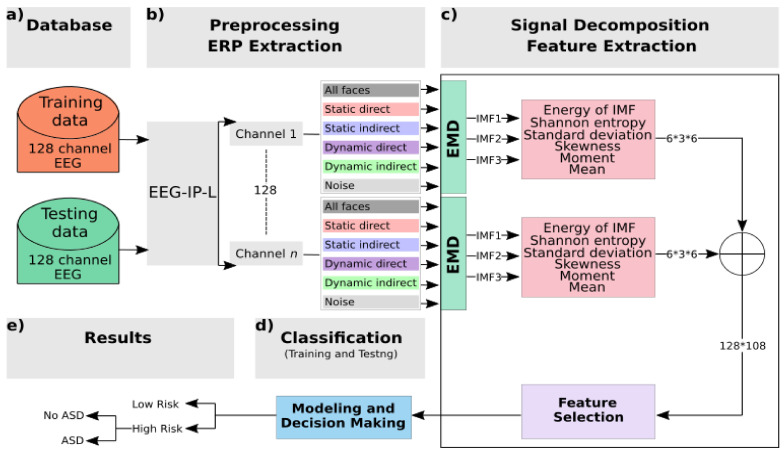
Block diagram of the system: (**a**) The BASIS database is split into training and testing using a nested cross-validation technique. (**b**) Preprocessing and ERP extraction: Raw EEG data were preprocessed using the EEG Integrated Platform (EEG-IP-L) pipeline [24]: all kinds of noises and artifacts are suppressed; then clean data are segmented to get fixed-length epochs. (**c**) Signal decomposition and feature extractions: empirical mode decomposition (EMD) is applied to decompose signals into intrinsic mode functions (IMFs); then features are extracted per channel and per IMF. A selection step is used to reduce the number of features. (**d**) Classification block: using nested cross validation, features selected are used as input to two classifiers, support vector machine (SVM) and *k*-nearest neighbors (*k*-NN), at the training and testing stages. (**e**) Class labels of data are predefined to distinguish familial risk, then diagnostic outcome within the high-risk group.

The process of establishing the classification system is generally composed of a training stage and a testing stage. In each of these stages, EMD and supervised machine learning were used to classify infants on the basis of familial risk and also by diagnostic outcome. The analysis framework is presented in Figure 2.

1. The EEG data were preprocessed to isolate and flag artifact for removal. The retained data were segmented into −200 to 800 ms epochs and averaged within condition to generate the ERP waveforms.
2. The single-channel ERP for each condition was decomposed to the first three IMFs using the EMD technique.
3. Energy and Shannon entropy were extracted along with four statistical parameters from each IMF: standard deviation, skewness, moment, and mean.
4. The maximum value, across all channels, for each of these six features was used in a selection step to determine the strongest features based on their weight correlation.
5. During the training stage, the vector of selected features that was extracted using an input signal from the training database was then fed into two classifiers to train and create models.
6. During the testing stage, the input vector chosen from the testing database was classified using trained models in order to associate the unknown input to one class.

Detailed steps are below.

#### 2.2.1. EEG Preprocessing

Raw EEG data were preprocessed using the EEG Integrated Platform (EEG-IP-L) pipeline [24], which includes a set of standardized and automated procedures to isolate cortical EEG signals from noise while maintaining maximal information from raw recordings and minimizing data loss. The output from the pipeline are continuous raw EEG data with comprehensive annotations regarding data quality and spatial nonstationarity. Compared with other standard automated procedures, the EEG-IP-L pipeline has been shown to increase data retention across subjects and single trials [24].

EEG-IP-L builds annotations regarding signal quality through a series of criterion functions that involve computing a metric (e.g., voltage variance) and building distributions in order to assess whether channels, time periods, or independent components are likely outliers on the given metric. As a first step, the signal properties of the scalp channels are examined. Continuous EEG is epoched in 1 s nonoverlapping windows. The standard deviation of the voltage across channels is calculated for each of these 1 s windows. A channel is flagged for the entire recording if, in more than 20% of the 1 s epochs, the voltage is more than six times the 0.3 to 0.7 inter-quantile range. Similarly, a 1 s epoch is flagged if more than 20% of the channels are outliers based on voltages that are more than six times the 0.3 to 0.7 inter-quantile range. The windowed data are concatenated back into the continuous time course, and a 1 Hz high-pass filter is applied in order to help establish a reliable Independent Component Analysis ICA decomposition, which is sensitive to nonstationary artifacts generated by large low-frequency oscillations (e.g., movement artifact and sweat artifacts). ICA decompositions have been shown to be more reliable when a high-pass filter is applied to the data (e.g., 1 Hz; [25]).

The next assessment of scalp signals is to identify channels that have unreliable activity or may be bridged with neighboring channels. Continuous data are windowed into 1 s nonoverlapping epochs, and the maximum correlation between each channel and its three spatially nearest neighbors is stored. A channel is flagged for the duration of the recording if, in more than 20% of the duration, it shows a maximum correlation that is six times less than 20% less the 0.3 to 0.7 inter-quantile range. Similarly, a 1 s epoch is flagged if more than 20% of the channels are outliers based on maximum neighbor correlation coefficients that are less than six times the 0.3 to 0.7 inter-quantile range. To identify bridged channels, a composite measure is created by taking the maximum correlation array and dividing the median by the interquartile range for each channel across time. This yields a value that accentuates high and invariable correlations across time. Channels are flagged as bridged if this composite value exceeds six standard deviations (40% trimmed) from the mean (40% trimmed) across the channels.

The windowed data are concatenated back into the continuous time course, and any channels or time periods that were not flagged are submitted to AMICA for decomposition. Similar to the procedures for scalp channels, following AMICA, the data are windowed into 1 s nonoverlapping epochs, and the standard deviation of Independent Components IC activations is calculated to determine time periods of relative nonstationarity. A 1 s epoch is flagged if more than 20% of ICs are outliers based on values that are more than six times the 0.3 to 0.7 inter-quantile range. Ignoring these time periods in which too many ICs have outlying voltage values, a subsequent AMICA is performed to generate a more reliable decomposition. 

A single dipole is fit to each IC weight topography. ICs are then classified into seven common categories (brain, eye, muscle, heart, channel noise, line noise, and other) using the ICLabel [26,27] in an EEGLAB extension. To classify ICs, the ICLabel extension examines the spatiotemporal measures in the ICLabel database, which contains more than 200,000 ICs sourced from over 6000 EEG recordings.

The final quality control was carried out by an expert review of the classification of ICs into the phenomena they capture paired with IC properties (e.g., topographical projection, spectral dynamics, dipole fit residual variance, and classification accuracy) and the comprehensive data annotations overlaid on the continuous time series of scalp and component activations. For a complete description of EEG-IP-L and a summary of data diagnostics, see [24,28].

#### 2.2.2. ERP Extraction

**Figure 3:**
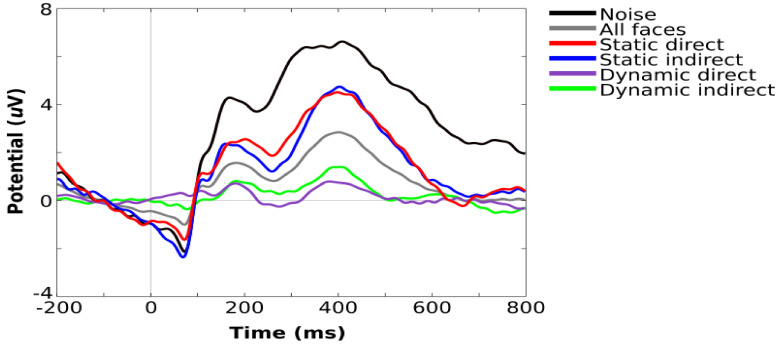
Grand average visual ERPs for all task conditions showing components typically observed during infant face processing, including P100, N290, and P400.

Scalp channels, time periods, and independent components that were flagged during preprocessing were purged from the data. Removed channels were interpolated using spherical spline, and all channels were then rereferenced to the average of all electrodes. Epochs were extracted (−200 to 800 ms), time-locked to stimulus onset, and baseline-corrected using the −200 to 0 window. Epochs were averaged within each stimulus condition to generate ERPs. Figure 3 shows the grand averaged visual ERPs for all task conditions from an occipital channel cluster that corresponds to those used in previous studies with the same sample [6,28].

#### 2.2.3. Signal Decomposition

To obtain useful features from the preprocessed ERP, EMD was applied to all single channels. It decomposes the signal into a set of intrinsic mode functions (IMFs) that reflect the oscillatory components of the signal, as well as a residual function using a sifting process. Specifically, after averaging over trials for each stimulus condition separately, EMD separates the signal into different frequency bands. If the signal is not contaminated with noise, then the first IMF corresponds to the highest frequency component.

This pattern repeats such that the second IMF corresponds to the next highest frequency component and the third IMF corresponds to the next highest frequency component. IMFs were extracted directly from the original ERP signal in the time domain while preserving the frequency variation in time. EMD has been widely used and applied to biomedical signals, particularly for nonlinear and nonstationary signals. EMD provides better time resolution than wavelet packet transforms and Fourier transform due to its instantaneous frequency property [29].

Each IMF must satisfy two basic criteria:

1. The original signal and the extracted IMF cannot differ by more than one with respect to the number of zero-crossing rates and extrema.
2. The mean value of the envelope representing the local maxima and the envelope representing the local minima must be zero at each time point.

To extract an IMF from an ERP waveform called *erp*(*t*) where 1…T, the following six steps of the sifting process are adopted:

1. Identify local maxima and local minima, so-called extrema, in the observed $erp\left(t\right)$
2. Generate the lower envelope by interpolating local minima $e_{min}\left(t\right)$
3. Generate the upper envelope by interpolating local maxima $e_{max}\left(t\right)$
4. Calculate the mean of the lower and upper envelopes as
  $$m\left(t\right)=\frac{e_{min}\left(t\right)+e_{max}\left(t\right)}{2}$$
5. Retrieve the detail from the original signal
  $$d\left(t\right)=erp\left(t\right)-m\left(t\right)$$

Test to see whether $d\left(t\right)$ satisfies an IMF’s basic criteria: if the criteria are satisfied, then the sifting is stopped, and *d*(*t*) is considered the first IMF and the original signal *erp*(*t*) is replaced with the residual *r*(*t*) = *erp*(*t*) − *d*(*t*).

The process is repeated k times until the first IMF is extracted; therefore,

$$imf_{1}\left(t\right)=d_{1k}\left(t\right)$$

and the residual signal:

$$r_{1}\left(t\right)=erp\left(t\right)-imf_{1}\left(t\right)$$

$r_{1}\left(t\right)$ is considered the new signal for the second sifting process from which the second IMF is extracted, and so on. The sifting process is repeated until the residual signal satisfies the stopping criteria.

At the end of the IMF extraction process, the original signal can be considered a linear combination of IMFs and the residual function, which can be expressed as follows:

$$erp\left(t\right)=\overset{n}{\underset{i=1}{\sum }}imf_{i}\left(t\right)+r_{n}\left(t\right)$$

After several initial tests, it was concluded that IMF4 and higher levels have smoother oscillations and do not contribute to our classification problems. Therefore, for the purposes of this work, the EMD of the ERP waveforms was achieved by computing IMF1, IMF2, and IMF3, and we considered only these features to assess the classification of familial risk and diagnostic outcome classifications.

#### 2.2.4. Feature Extraction and Selection

Effective features need to be extracted to represent original input signals and to be input vectors for the subsequent classifier. In this step, the time series of each single channel IMF are treated separately for each ERP condition. Six features were calculated for each IMF: IMF energy, Shannon entropy, and four statistical parameters (mean, standard deviation, skewness, and moment) extracted directly from the IMF in the discrete time domain.

1. IMF energy describes the weight of oscillation. It provides a quantitative measure of the strength of the oscillation over a finite period. It is defined as the sum of squared absolute values of IMFs and is calculated as follows:
  $$E_{IMF_{\left(c,i\right)}}=\overset{N}{\underset{k=1}{\sum }}IMF_{\left(c,i\right)}^{2}\left(k\right)$$
2. Shannon entropy describes the measure of the impurity or the complexity of the time series signal. The concept is introduced in “A mathematical theory of communication” (1948) by Shannon. In this study, Shannon entropy is computed for each IMF to evaluate its complexities. The formula of entropy calculation is as follows:
  $$SE_{IMF_{\left(c,i\right)}}=-\overset{N}{\underset{k=1}{\sum }}IMF_{\left(c,i\right)}^{2}\left(k\right)\times logIMF_{\left(c,i\right)}^{2}\left(k\right)$$
3. Four statistical parameters were derived from IMF signals: mean, standard deviation, skewness, and moment. These parameters were extracted directly from each IMF per channel to represent its statistical distribution.

After the extraction step, the six features were combined to form 128 (one per channel) hybrid vectors of three dimensions (*i*, *j*, *k*). Here, *i* is equal to 6 and represents the number of visual tasks, *j* is equal to 3 and represents the number of IMFs, and *k* is equal to 6 and represents the number of features extracted from each IMF. Hence, for each input we obtained 108 features. Considering the 128 channels, the output feature vector is sized 128 × 108. A selection step based on the maximum values across all channels was deployed to decrease the vector dimension to 1 row and 108 columns. When features extracted from all IMFs and all condition ERPs were to be tested, an additional selection by weight correlation was performed to determine the top predictive features to be used as inputs to classifiers. All steps of ERP feature extraction were performed using MATLAB and EEGLAB [30] with the integrated plugin ERPLAB [31].

#### 2.2.5. Classification: Modeling and Decision-Making

The association between the features extracted and risk/diagnostic outcome is the main goal of the classification stage. The classification stage consists in assigning given input data into one of the classes when attempting to classify control vs. HR for risk status and HR-ASD vs. HR-noASD for diagnostic outcomes.

In this study, we analyzed the accuracy rate and performance of *k*-nearest neighbors (*k*-NN) and support vector machine (SVM).

The *k*-NN method is one of the simplest machine learning methods and consists in assigning unknown input samples to the class of the closest samples in the training set [32]. The distance between a new feature vector and all training vectors is computed. In the current study, Euclidean distance was used to measure the distance between a new unknown input and the trained samples. SVM, proposed by [33], consists in finding a hyperplane or boundary between two classes using their labels in a way that maximizes the separation margin between the classes. SVM has been used in various fields of research, including contexts in which the training dataset is small, and the number of features is low. Linear and nonlinear SVM classifiers were also considered in this study. Given that our two classification methods belong to the category of supervised learning, we predefined class labels of data for familial risk and diagnostic outcome. During the training stage, the feature input vectors were fed into the classifier, which, in turn, adjusted its variable parameters to capture the relationship between the input and the predefined classes. The aim of this stage is to build a model for each group in each of the two classification problems (risk and outcome). This model can be used to test a new input in one of the given groups. In our case, we performed a large battery of tests with the classifiers that included feeding extracted features into *k*-NN and SVM separately in order to classify inputs. In addition, for comparison purposes, we studied the performance of classifiers per condition ERP to examine which stimulus category yields the most adequate information and which IMFs best represent the ERP.

#### 2.2.6. Performance Evaluation

The effectiveness of the proposed framework is studied by computing three parameters: accuracy rate, sensitivity, and specificity.

Accuracy represents the measure of the classifiers’ ability to correctly classify groups, and it is calculated using the following equation:

$$AccuracyRate=\frac{Nboftruepositives+Nboftruenegatives}{Nboftruepositives+Nboftruenegatives+Nboffalsepositives+Nboffalsenegatives}$$

The number of true positives represents the number of subjects correctly classified as HR in the first experiment and HR-ASD in the second experiment (called positive records). The number of true negatives is the number of subjects correctly classified as control in the first experiment or HR-noASD in the second experiment (known as negative records). The number of false positives is the number of subjects incorrectly classified as HR in the first experiment and HR-ASD in the second experiment. The number of false negatives is the number of subjects incorrectly classified as control in the first experiment or HR-noASD in the second experiment. 

Sensitivity represents the classifiers’ ability to correctly classify positive records, and it is calculated using the following equation:

$$Sensitivity=\frac{numberoftruepositives}{numberoftruepositives+numberoffalsenegatives}$$

Specificity represents the classifiers’ ability to correctly classify negative records, and it is calculated as follows:

$$Specificity=\frac{numberoftruenegatives}{numberoftruenegatives+numberoffalsepositives}$$

For validation, the data were split into two training and testing datasets using nested cross validation. An outer k-fold cross validation was used to split the data into k subsets (k-1 subsets for training and one subset for testing) and to produce the final classification accuracy estimates. An inner loop of k-fold cross validation was run on each training fold to select the optimal model based on the best hyperparameters (number of k neighbors for *k*-NN classifier and parameters C and gamma to optimize classification using SVM). The classification process is repeated k times. For each iteration, k-1 subsets are employed for training and one subset for testing the model. Parameters of performance are computed based on the outer test folds.

## 3. Results

We processed the data in two steps: First, we performed a set of experiments focused on the modelling and classification of participants in the control and HR groups. Second, we focused on the modelling and classification of diagnosis outcomes within the high-risk group: HR-ASD and HR-noASD. The study sample comprised 44 control and 50 HR 6-month-old infants. EEG signals were divided into six averaged ERPs that represented the six stimulus conditions (Figure 1). The total number of 1 s segments from each participant across the six stimulus conditions resulted in 564 observations used in the classification of HR and control groups. Given the smaller sample size of those who went on to develop autism (17 HR-ASD infants) vs. those who did not (33 HR-noASD infants), and to equate sample sizes and minimize overfitting bias, we performed a resampling approach, whereby subsets of 17 subjects were randomly selected (without replacement) from the 33 HR-noASD infants. Thirty-four EEG recordings were included in each iteration, 17 HR-ASD and 17 HR-noASD, for a total of 204 observations used in the group classification. This resampling process was repeated five times. The results of the classifiers *k*-NN and SVM for each classification problem were studied separately (Table 2 and Table 3). Comparisons between classifiers are presented in Table 4. To get an unbiased performance estimate, testing data were not used to optimize models, including feature selection. Therefore, the available dataset was divided into training and testing using nested cross validation: 9 × 10 (number of inner folds × number of outer folds) for the first classification problem (HR vs. control). A 4 × 5 cross validation was used in the second classification problem (HR-ASD and HR-noASD) due to the lower number of samples used.

### 3.1. Classification of HR vs. Control Using k-NN

**Figure 4:**
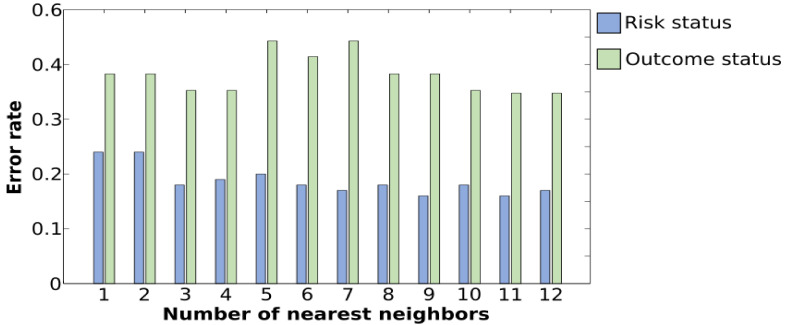
Plot of error rates of the testing set for different values of *k*. A lower error rate in the testing stage is obtained with *k* = 11 for the classification of the diagnosis outcome as well as for the classification of risk status.

For the classification of the risk groups, an accuracy rate of 86.22% was achieved for the *k*-NN classifier when the top 11 features selected by weight correlation were used as inputs to the classifier (see Table 2 for detailed results). IMF1, IMF2, and IMF3 correspond to the use of one component at a time. Thus, IMF1–3 represents the use of IMF1, IMF2, and IMF3 together. The sensitivity measure, which indicates the ability of the *k*-NN classifier to correctly identify high-risk samples, reached a maximum of 80.00%, while the specificity measure, which indicates the ability of the *k*-NN classifier to correctly identify low-risk samples, reached a maximum of 93.18%. Table 5 represents the 10 features that contribute the most to the prediction of familial risk. The skewness of the third IMF that represents the averaged ERP epoch during the averted gaze stimuli is on the strongest feature. The other nine ranked features are statistical parameters, except the seventh, which is the Shannon entropy of the first IMF extracted during direct gaze stimuli. Different runs were executed by varying the number of nearest neighbors from which to choose the best value of k. This process, called parameter tuning, is critical in order to obtain a better classification accuracy; Figure 4 shows that the lowest error rates are obtained when k equals 11. A comparison of system performances across the six stimulus categories is presented in Table 2. Direct gaze stimuli showed the strongest discriminative features among all stimulus categories in terms of correctly classifying HR infants with an accuracy rate of 76.60%, a sensitivity of 72.00%, and a specificity of 82.00%. Direct gaze was followed by averted gaze, which had an accuracy of 73.40%. To check the order of importance of IMFs, another set of experiments was performed where *k*-NN classification was completed using features extracted from one IMF at a time (Table 2). A maximum accuracy rate of 77.70% was obtained while employing IMF2 with a significant sensitivity of 80.00% and a specificity of 75.00%.

### 3.2. Classification of HR and Control Using SVM

**Figure 5:**
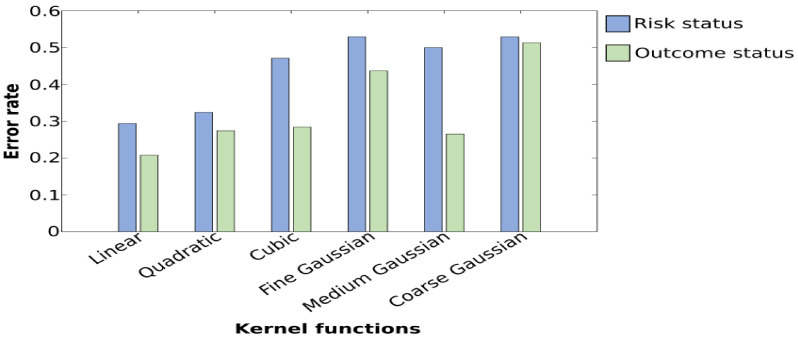
Plot of error rates of the testing set obtained while employing different kernel functions. Graphs show that for the classification of the risk group and outcome status, a minimal error rate is obtained with linear SVM. A maximal error rate is obtained with coarse Gaussian SVMs when classifying risk status and with the fine Gaussian and coarse Gaussian when classifying the diagnosis outcome.

Table 2 summarizes the classification performance using SVM. For the classification risk, an accuracy rate of 88.44% with a sensitivity of 84.00% was achieved for the SVM classifier when the top 30 features, selected by weight correlation, were used as inputs to the classifier. It is worth noting that the selection by weight correlation was done using the training data. Figure 5 shows a comparison between linear and nonlinear SVMs and indicates that the minimal error rate was obtained with a linear SVM. The performance of the SVM classifier in classifying HR and control groups in terms of accuracy, sensitivity, and specificity was compared across the different stimulus conditions. Table 2 shows that a maximal accuracy rate of 77.70% was obtained for the direct gaze stimulus with a significant sensitivity of 82.00% and a specificity of 73.00%. To determine the most suitable IMF, the performance was again examined for each IMF separately. We found that IMF2 had the most discriminative information with an accuracy of 80.90% and a sensitivity of 78.00%. Comparing this finding with the same classification problem obtained using *k*-NN, we conclude that the importance of IMF2 is highest among all IMFs for both classifiers.

### 3.3. Classification of HR-ASD and HR-noASD Using k-NN

For the classification of diagnostic outcome in high-risk infants, an accuracy rate of 74.00% was achieved for the *k*-NN classifier when the top 11 features, selected by weight correlation, were used as inputs to the classifier (Table 3).

Feature relevance is presented in Table 6, which shows that the skewness of IMFs is one of the strongest predictors among all features.

We also examined the performance of the *k*-NN classifier in terms of accuracy, sensitivity, and specificity as a function of the stimulus condition. In each experiment on the classification of diagnostic outcome groups, features extracted from each of the ERP conditions were fed into the *k*-NN classifier one at a time. A maximal accuracy rate of 73.50% was obtained for the noise stimulus condition (see Table 3 and Table 6). The two most important features that contributed to the highest prediction were derived from the noise stimuli; however, there was only 53.00% sensitivity in correctly classifying HR-ASD, which is low and inadequate. To check the suitability of each IMF, a set of experiments was done to study the performance of *k*-NN when used along with features extracted from one IMF at a time. The results in Table 3 show that percentages are low compared with the use of all features and all IMFs combined. The maximal accuracy rate was obtained with IMF1, whereas the best sensitivity was obtained with IMF3.

### 3.4. Classification of HR-ASD and HR-noASD Using SVM 

For the classification of HR-ASD and HR-noASD using SVM, the highest accuracy rate obtained was 70.48%; however, this was associated with a lower specificity of 64.71% compared with 70.00% derived from *k*-NN. Overall, accuracy rates were low across approaches in regard to stimulus condition and the level of importance of IMFs. A maximal value of 67.60% was obtained for the noise condition, with a sensitivity of 76.00% and a specificity of 59.00% (Table 3). Further, these results indicate a maximal value of 67.60% with features extracted from IMF1 and a sensitivity of 59.00%, indicating that SVM is insufficient for the classification of diagnostic outcome. Moreover, despite multiple trial runs to improve the performance of SVM by varying kernel functions, the results show that a lower error rate is consistently obtained with linear SVM (Figure 5).

## 4. Discussion

In this study, we examined whether the integration of machine learning with EMD for advanced ERP processing can be used to classify 6-month-old infants in terms of familial risk for ASD (at risk vs. control) and diagnostic outcomes (ASD vs. no ASD). Previous work has focused on using the EEG frequency domain, time domain, or time–frequency domains [8,14,34,35,36]. In the current study, we used a machine learning approach based on features extracted in the EMD domain derived from ERPs during a face and gaze processing task. Both SVM and *k*-NN methods distinguished infants according to familial risk and diagnostic outcomes. Using nested cross validation and feature selection by weighted correlation, SVM achieved a maximal accuracy rate of 88.44% in the classification of at-risk infants from controls based on EMD features. This classification yields a 24.44% increase in performance compared with that in previous work using a standard time domain of ERP analysis [19]. Our findings suggest that extracting signal features based on an EMD approach (from the same data) can enhance classification performance compared with traditional ERP features (amplitude and peak latency), thus making EMD an innovative technique for the analysis of nonstationary time series signals.

In contrast to the relatively high accuracy of classification by familial risk, we found that rates are more modest for classification by diagnostic outcomes. A maximum accuracy of 74.00% for the classification of HR infants who went on to develop ASD from those who did not was obtained using *k*-NN, but not SVM. Further, *k*-NN performs well even with a small sample size of training data; therefore, the lower classification accuracy for diagnostic outcome compared with familial risk is likely due to the lower number of training samples, valid trials, and variability in responses among infants. Further research with larger pooled samples is needed to differentiate these possibilities.

In previous works, statistics-based features of IMFs have been widely used and have been shown to be effective in different classification problems, such as sleep vs. wake stages, discrimination between normal and pathological EEG signals, and detection of seizure and epilepsy [20,37,38,39]. Their use is justified by the fact that the sampling distribution of a given population is defined by its statistical dispersion, distortion, or asymmetry. In addition, signal energy and complexity have been widely employed to analyze EEG signals [20,22,39]. In this study, we chose to use different combinations of the extracted features to study the performance of classifiers per single IMF and per task condition. We investigated the role of each IMF component by studying the performance of classifiers with features extracted from one IMF at a time. Our findings demonstrate that, overall, IMFs can be considered good candidates for analyzing nonstationary ERP signals. Further, we found that IMF1, the highest frequency component, yielded the best classification by diagnostic outcome compared with IMF2 and IMF3. In addition, IMF2, the second highest frequency component, was a better indicator for classification by familial risk. Thus, we found that using features extracted from one single IMF yielded a lower performance compared with using features from all IMFs combined.

Previous studies have shown that beta and gamma bands are found within IMF1, IMF2, and IMF3 derived from EEG signals [37,38]. High-frequency oscillatory activity within beta and gamma bands has also been associated with ASD risk and outcomes [2,40]. Our findings are in line with these previous studies as they show that IMF1, IMF2, and IMF3 are associated with activity in these frequency bands and are strong predictors of ASD risk and diagnostic outcome [2,39] and that higher levels of signal decomposition beyond IMF3 (which reflect lower frequency oscillations) did not enhance classification rates. In addition, we found that the classification accuracy of the risk group was highest when using features from the direct gaze condition, which was similarly shown in previous studies, indicating that ERPs to direct gaze stimuli distinguished high risk from control infants [2,6,19].

## 5. Conclusions

In previous research, EMD has been successfully used for emotional state recognition from EEG signals; epileptic seizure detection; discrimination between ictal, interictal, and normal EEG signals; classification of seizure and seizure-free EEG signals; and sleep state detection [20,21,22,23,37,38]. Our findings suggest that EMDs derived from ERPs, coupled with machine learning techniques, could contribute to the development of identifying early brain indicators of ASD and typical development. Our approach differs from previous methods as it is based on features extracted from the EMD domain to decompose ERP waveforms into a set of IMFs.

Only a binary classification problem was considered in this study due to the limited sample size. Larger groups of participants, particularly those who are at familial risk and go on to have ASD diagnosis, are needed to consider a three-class discrimination between control, HR-noASD, and HR-ASD. Future studies will also benefit from a more fine-grained analysis of single EEG trials in addition to features extracted from a combination of IMFs of the averaged ERP. Furthermore, simultaneous analyses of features extracted from the EMD decomposition of both ERP and resting-stage EEG may allow us to increase the performance of machine learning methods in the prediction of risk and outcome.

## Figures and Tables

**Table 1:** The number of participants available for analysis from each group: high-risk infants who did not receive a diagnosis (HR-noASD), high-risk infants who received a diagnosis (HR-ASD).

	Control	HR-noASD	HR-ASD	All
Male	15 (43%)	9 (26%)	11 (31%)	35 (37%)
Female	29 (49%)	24 (41%)	6 (10%)	59 (63%)
Total	44 (47%)	33 (35%)	17 (18%)	94 (100%)

**Table 2:** For the classification of high-risk and low-risk groups: comparison between *k*-NN and SVM classifiers of accuracy rates, specificity, and sensitivity. The first six rows represent the results of analysis of the six stimulus conditions separately. Rows 7, 8, and 9 show the classification performance when using features extracted from one IMF at a time. Row 10 represents the results of analyzing the six stimulus conditions together with the IMFs when features were selected by weight correlation and used as input to classifiers: using *k*-NN, the best performance of 86.22% is obtained with 11 features, and using SVM, the best performance of 88.44% is obtained with top 30 features.

			Classification of HR and Control					
Condition	Component	Number of Features after Reduction	*k*-NN Performance			SVM Performance		
			Accuracy Rate	Sensitivity	Specificity	Accuracy Rate	Sensitivity	Specificity
Direct gaze	IMF1–3	18	76.60%	72.00%	82.00%	77.70%	82.00%	73.00%
Averted gaze	IMF1–3	18	70.20%	74.00%	66.00%	74.50%	76.00%	73.00%
Static direct	IMF1–3	18	60.60%	68.00%	52.00%	62.80%	68.00%	57.00%
Static averted	IMF1–3	18	73.40%	68.00%	80.00%	74.50%	86.00%	61.00%
Face	IMF1–3	18	71.30%	72.00%	70.00%	74.50%	78.00%	70.00%
Noise	IMF1–3	18	68.10%	72.00%	64.00%	63.80%	72.00%	55.00%
All	IMF1	36	63.80%	56.00%	73.00%	68.10%	68.00%	68.00%
All	IMF2	36	77.70%	80.00%	75.00%	80.90%	78.00%	84.00%
All	IMF3	36	74.50%	76.00%	73.00%	76.60%	68.00%	86.00%
All	IMF1–3	11	**86.22%**	80.00%	93.18%	-	-	-
		30	-	-	-	**88.44%**	84.00%	93.18%

**Table 3:** For the classification of the diagnosis outcome: comparison between *k*-NN and SVM classifiers of accuracy rates, specificity, and sensitivity. The first six rows represent the results of the analysis of the six stimulus conditions separately. Rows 7, 8, and 9 show the classification performance when using features extracted from one IMF at a time. Row 10 represents the results of analysis of the six stimulus conditions together with the IMFs with 11 selected features by weight correlation. Best accuracies of 74% and 70.48% were obtained using k-NN and SVM respectively.

			Classification of HR-ASD and HR-noASD					
Condition	Component	Number of Features after Reduction	*k*-NN Performance			SVM Performance		
			Accuracy Rate	Sensitivity	Specificity	Accuracy Rate	Sensitivity	Specificity
Direct gaze	IMF1–3	18	64.70%	53.00%	76.00%	64.70%	53.00%	76.00%
Averted gaze	IMF1–3	18	47.10%	29.00%	65.00%	50.00%	41.00%	59%
Static direct	IMF1–3	18	55.90%	59.00%	53.00%	67.60%	71.00%	65.00%
Static averted	IMF1–3	18	55.90%	65.00%	47.00%	50.00%	47.00%	53.00%
Face	IMF1–3	18	52.90%	29.00%	76.00%	55.90%	53.00%	59.00%
Noise	IMF1–3	18	73.50%	53.00%	94.00%	67.60%	76.00%	59.00%
All	IMF1	36	64.70%	47.00%	82.00%	67.60%	59.00%	76.00%
All	IMF2	36	47.10%	47.00%	47.00%	58.80%	53.00%	65.00%
All	IMF3	36	55.90%	53.00%	59.00%	61.80%	47.00%	76.00%
All	IMF1–3	11	**74.00%**	78.00%	70.00%	**70.48%**	76.47%	64.71%

**Table 4:** Best results obtained for all experiments of this study for both HR vs. control and HR-ASD vs. HR-noASD classifications: the names of the best classifiers are presented along with their accuracy rate and the number of features used in each experiment.

			HR vs. Control		HR-ASD vs. HR-noASD	
Condition	Component	Number of Features	Best Classifier	Accuracy Rate	Best Classifier(s)	Accuracy Rate
Direct gaze	IMF1–3	18	SVM	77.7	*k*-NN and SVM	64.70
Averted gaze	IMF1–3	18	SVM	74.5	SVM	50.00
Static direct	IMF1–3	18	SVM	62.8	SVM	67.60
Static averted	IMF1–3	18	SVM	74.50	*k*-NN	55.90
Face	IMF1–3	18	SVM	74.50	SVM	55.90
Noise	IMF1–3	18	*k*-NN	68.10	*k*-NN	73.50
All	IMF1	36	SVM	68.1	SVM	67.60
All	IMF2	36	SVM	80.9	SVM	58.80
All	IMF3	36	SVM	76.6	SVM	61.80
All	IMF1–3	30/11	SVM	88.44	*k*-NN	74.00

**Table 5:** Ranked predictor importance for the *k*-NN classifier predicting familial risk from six features and six averaged epochs.

Prediction Importance Target: Familial Risk			
Rank	Feature	IMF#	Stimulus
1	Skewness	IMF3	Averted gaze
2	Skewness	IMF1	Face
3	Std	IMF2	Static direct
4	Std	IMF1	Noise
5	Std	IMF2	Face
6	Skewness	IMF2	Direct gaze
7	Shannon entropy	IMF1	Direct gaze
8	Std	IMF2	Static averted
9	Mean	IMF2	Noise
10	Moment	IMF1	Noise

**Table 6:** Predictor importance for the *k*-NN classifier predicting diagnostic outcome from six features and six averaged epochs.

Prediction Importance Target: HR-ASD and HR-noASD			
Rank	Feature	IMF#	Stimulus
1	Skewness	IMF2	Noise
2	Skewness	IMF3	Noise
3	Energy	IMF2	Static direct
4	Shannon entropy	IMF1	Static direct
5	Moment	IMF3	Static direct
6	Skewness	IMF3	Static direct
7	Skewness	IMF1	Static averted
8	Skewness	IMF3	Averted gaze
9	Skewness	IMF3	Static averted
10	Skewness	IMF1	Static direct

## Data Availability

Not applicable.

## References

[1] Ozonoff S., Young G.S., Carter A., Messinger D., Yirmiya N., Zwaigenbaum L., Bryson S., Carver L.J., Constantino J.N., Dobkins K. (2011). Recurrence Risk for Autism Spectrum Disorders: A Baby Siblings Research Consortium Study. Pediatrics.

[2] Elsabbagh M., Volein A., Csibra G., Holmboe K., Garwood H., Tucker L., Krljes S., Baron-Cohen S., Bolton P., Charman T. (2009). Neural Correlates of Eye Gaze Processing in the Infant Broader Autism Phenotype. Biol. Psychiatry.

[3] Jeste S.S., Frohlich J., Loo S.K. (2015). Electrophysiological biomarkers of diagnosis and outcome in neurodevelopmental disorders. Curr. Opin. Neurol..

[4] Bussu G., Jones E.J.H., Charman T., Johnson M.H., Buitelaar J.K., Baron-Cohen S., Bedford R., Bolton P., Blasi A., Chandler S. (2018). Prediction of Autism at 3 Years from Behavioural and Developmental Measures in High-Risk Infants: A Longitudinal Cross-Domain Classifier Analysis. J. Autism Dev. Disord..

[5] O’Reilly C., Lewis J.D., Elsabbagh M. (2017). Is functional brain connectivity atypical in autism? A systematic review of EEG and MEG studies. PLoS ONE.

[6] Elsabbagh M., Mercure E., Hudry K., Chandler S., Pasco G., Charman T., Pickles A., Baron-Cohen S., Bolton P., Johnson M.H. (2012). Infant neural sensitivity to dynamic eye gaze is associated with later emerging autism. Curr. Biol..

[7] Dawson G., Carver L., Meltzoff A.N., Panagiotides H., McPartland J., Webb S.J. (2002). Neural correlates of face and object recognition in young children with autism spectrum disorder, developmental delay, and typical development. Child Dev..

[8] McPartland J., Dawson G., Webb S.J., Panagiotides H., Carver L.J. (2004). Event-related brain potentials reveal anomalies in temporal processing of faces in autism spectrum disorder. J. Child Psychol. Psychiatry Allied Discip..

[9] Luck S.J. (2005). An Introduction to Event-Related Potentials and Their Neural Origins. Introd. Event-Relat. Potential Tech..

[10] McCleery J.P., Akshoomoff N., Dobkins K.R., Carver L.J. (2009). Atypical Face Versus Object Processing and Hemispheric Asymmetries in 10-Month-Old Infants at Risk for Autism. Biol. Psychiatry.

[11] Key A.P.F., Stone W.L. (2012). Processing of novel and familiar faces in infants at average and high risk for autism. Dev. Cogn. Neurosci..

[12] Zhang F., Savadjiev P., Cai W., Song Y., Rathi Y., Tunç B., Parker D., Kapur T., Schultz R.T., Makris N. (2018). Whole brain white matter connectivity analysis using machine learning: An application to autism. NeuroImage.

[13] Usta M.B., Karabekiroglu K., Sahin B., Aydin M., Bozkurt A., Karaosman T., Aral A., Cobanoglu C., Kurt A.D., Kesim N. (2019). Use of machine learning methods in prediction of short-term outcome in autism spectrum disorders. Psychiatry Clin. Psychopharmacol..

[14] Bosl W.J., Tager-Flusberg H., Nelson C.A. (2018). EEG Analytics for Early Detection of Autism Spectrum Disorder: A data-driven approach. Sci. Rep..

[15] Grossi E., Olivieri C., Buscema M. (2017). Diagnosis of autism through EEG processed by advanced computational algorithms: A pilot study. Comput. Methods Programs Biomed..

[16] Jamal W., Das S., Oprescu I.A., Maharatna K., Apicella F., Sicca F. (2014). Classification of autism spectrum disorder using supervised learning of brain connectivity measures extracted from synchrostates. J. Neural Eng..

[17] Bone D., Bishop S.L., Black M.P., Goodwin M.S., Lord C., Narayanan S.S. (2016). Use of machine learning to improve autism screening and diagnostic instruments: Effectiveness, efficiency, and multi-instrument fusion. J. Child Psychol. Psychiatry Allied Discip..

[18] Bosl W., Tierney A., Tager-Flusberg H., Nelson C. (2011). EEG complexity as a biomarker for autism spectrum disorder risk. BMC Med..

[19] Stahl D., Pickles A., Elsabbagh M., Johnson M.H. (2012). Novel machine learning methods for ERP analysis: A validation from research on infants at risk for autism. Dev. Neuropsychol..

[20] Martis R.J., Acharya U.R., Tan J.H., Petznick A., Yanti R., Chua C.K., Ng E.Y.K., Tong L. (2012). Application of empirical mode decomposition (EMD) for automated detection of epilepsy using EEG signals. Int. J. Neural Syst..

[21] Pachori R.B. (2008). Discrimination between Ictal and Seizure-Free EEG Signals Using Empirical Mode Decomposition. Res. Lett. Signal Process..

[22] Sharma R., Pachori R.B. (2015). Classification of epileptic seizures in EEG signals based on phase space representation of intrinsic mode functions. Expert Syst. Appl..

[23] Fu K., Qu J., Chai Y., Dong Y. (2014). Classification of seizure based on the time-frequency image of EEG signals using HHT and SVM. Biomed. Signal Process. Control.

[24] Desjardins J.A., van Noordt S., Huberty S., Segalowitz S.J., Elsabbagh M. (2021). EEG Integrated Platform Lossless (EEG-IP-L) pre-processing pipeline for objective signal quality assessment incorporating data annotation and blind source separation. J. Neurosci. Methods.

[25] Winkler I., Debener S., Muller K.R., Tangermann M. (2015). On the influence of high-pass filtering on ICA-based artifact reduction in EEG-ERP. Proceedings of the Annual International Conference of the IEEE Engineering in Medicine and Biology Society, EMBS.

[26] Pion-Tonachini L., Kreutz-Delgado K., Makeig S. (2019). The ICLabel dataset of electroencephalographic (EEG) independent component (IC) features. Data Brief.

[27] Pion-Tonachini L., Kreutz-Delgado K., Makeig S. (2019). ICLabel: An automated electroencephalographic independent component classifier, dataset, and website. NeuroImage.

[28] van Noordt S., Desjardins J.A., Huberty S., Abou-Abbas L., Webb S.J., Levin A.R., Segalowitz S.J., Evans A.C., Elsabbagh M. (2020). EEG-IP: An international infant EEG data integration platform for the study of risk and resilience in autism and related conditions. Mol. Med..

[29] Huang N.E., Szu H.H., Vetterli M., Campbell W.J., Buss J.R. (2000). New method for nonlinear and nonstationary time series analysis: Empirical mode decomposition and Hilbert spectral analysis. Proceedings of the Wavelet Applications VII.

[30] Delorme A., Makeig S. (2004). EEGLAB: An open source toolbox for analysis of single-trial EEG dynamics including independent component analysis. J. Neurosci. Methods.

[31] Lopez-Calderon J., Luck S.J. (2014). ERPLAB: An open-source toolbox for the analysis of event-related potentials. Front. Hum. Neurosci..

[32] Cover T.M., Hart P.E. (1967). Nearest Neighbor Pattern Classification. IEEE Trans. Inf. Theory.

[33] Cortes C., Vapnik V. (1995). Support-vector networks. Mach. Learn..

[34] Behnam H., Sheikhani A., Mohammadi M.R., Noroozian M., Golabi P. Analyses of EEG background activity in Autism disorders with fast Fourier transform and short time Fourier measure. Proceedings of the 2007 International Conference on Intelligent and Advanced Systems, ICIAS 2007.

[35] Subha D.P., Joseph P.K., Acharya U.R., Lim C.M. (2010). EEG signal analysis: A survey. J. Med. Syst..

[36] Sheikhani A., Behnam H., Mohammadi M.R., Noroozian M., Mohammadi M. (2012). Detection of abnormalities for diagnosing of children with autism disorders using of quantitative electroencephalography analysis. J. Med. Syst..

[37] Alam S.M.S., Bhuiyan M.I.H. (2013). Detection of seizure and epilepsy using higher order statistics in the EMD domain. IEEE J. Biomed. Health Inform..

[38] Orosco L., Laciar E., Correa A.G., Torres A., Graffigna J.P. An epileptic seizures detection algorithm based on the empirical mode decomposition of EEG. Proceedings of the 31st Annual International Conference of the IEEE Engineering in Medicine and Biology Society: Engineering the Future of Biomedicine, EMBC 2009.

[39] Oweis R.J., Abdulhay E.W. (2011). Seizure classification in EEG signals utilizing Hilbert-Huang transform. Biomed. Eng. Online.

[40] Tierney A.L., Gabard-Durnam L., Vogel-Farley V., Tager-Flusberg H., Nelson C.A. (2012). Developmental trajectories of resting eeg power: An endophenotype of autism spectrum disorder. PLoS ONE.

